# Seroprevalence of antibodies against SARS-CoV-2 in the school community in Campo Grande, state of Mato Grosso do Sul, Brazil, October 2021–November 2022

**DOI:** 10.3389/fimmu.2024.1354786

**Published:** 2024-03-26

**Authors:** Claudia Stutz, Daniel Maximo Correa Alcantara, Camila Maria dos Santos, Jaire Marinho Torres, Rudielle Rodrigues, Glaucia Elisete Barbosa Marcon, Eduardo de Castro Ferreira, Elaine Cristina Fernandes Baez Sarti, Thiago Fernandes de Oliveira, Flavia Maria Lins Mendes, Everton Ferreira Lemos, Luiz Henrique Ferraz Demarchi, Gislene Garcia de Castro Lichs, Marina Castilhos Souza Umaki Zardin, Crhistinne Cavalheiro Maymone Gonçalves, Jislaine de Fátima Guilhermino, Renata Trentin Perdomo, Zoraida del Carmen Fernandez

**Affiliations:** ^1^ Fiocruz Ceará, Fundação Oswaldo Cruz (Fiocruz), Eusébio, Ceará, Brazil; ^2^ Programa de Pós-Graduação em Ciências Farmacêuticas, Faculdade de Ciências Farmacêuticas, Alimentos e Nutrição (FACFAN), Fundação Universidade Federal de Mato Grosso do Sul (UFMS), Campo Grande, Mato Grosso do Sul, Brazil; ^3^ Fiocruz Mato Grosso do Sul, Fundação Oswaldo Cruz (Fiocruz), Campo Grande, Mato Grosso do Sul, Brazil; ^4^ Programa de Pós-graduação em Doenças Infecciosas e Parasitárias, Faculdade de Medicina (FAMED), Fundação Universidade Federal de Mato Grosso do Sul (UFMS), Campo Grande, Mato Grosso do Sul, Brazil; ^5^ Instituto Integrado de Saúde (INISA), Fundação Universidade Federal de Mato Grosso do Sul (UFMS), Campo Grande, Mato Grosso do Sul, Brazil; ^6^ Universidade Estadual de Mato Grosso do Sul (UEMS), Campo Grande, Mato Grosso do Sul, Brazil; ^7^ Laboratório Central de Saúde Pública do Estado de Mato Grosso do Sul (LACEN-MS), Campo Grande, Mato Grosso do Sul, Brazil; ^8^ Secretaria de Estado de Saúde de Mato Grosso do Sul, Secretaria Adjunta de Estado, Campo Grande, Mato Grosso do Sul, Brazil; ^9^ Laboratório de Biologia Molecular e Cultura Celular da Faculdade de Ciências Farmacêuticas, Alimentos e Nutrição (FACFAN), Fundação Universidade Federal de Mato Grosso do Sul (UFMS), Campo Grande, Mato Grosso do Sul, Brazil

**Keywords:** COVID-19, serological survey, antibodies, educational institutions, children, adolescents

## Abstract

**Introduction:**

With the reopening of schools during the coronavirus disease 2019 (COVID-19) pandemic, it was imperative to understand the role of students and education professionals in the spread of severe acute respiratory syndrome coronavirus 2 (SARS-CoV-2). In this paper, we determined the seroprevalence of the SARS-CoV-2 anti-nucleocapsid antibodies in the school community in Campo Grande, the capital and most populous city of the state of Mato Grosso do Sul (Brazil) and evaluated its association with sex, school level, and school type.

**Materials and methods:**

The survey was carried out in 20 public and private schools in the urban region of Campo Grande using the TR DPP^®^ COVID-19 immunoglobulin M/immunoglobulin G (IgM/IgG) kit from the Immunobiological Technology Institute (Bio-Manguinhos, Rio de Janeiro, Brazil). Testing was carried out in three periods: from October to December 2021; from March to July 2022; and from August to November 2022. The participants were students aged 6–17 years enrolled in primary or secondary schools and professionals of different ages and roles.

**Results:**

During the first testing period, 162 participants were seropositive for the IgM and/or IgG anti-nucleocapsid SARS-CoV-2 antibodies, with an estimated seroprevalence of 19.6% using Bayesian multilevel regression. In the second period, 251 participants were seropositive (estimated seroprevalence, 34.6%), while in the third period, 393 participants were seroconverted (estimated seroprevalence, 56.7%). In 2022, there was an increase in the seroconversion rate compared to that in 2021. The most frequently described acute manifestations in the three periods were fever, headache, sore throat, and runny nose. In terms of the demographic profile, there was no predominance of seropositivity between the sexes, although women represented approximately 70% of the study population. There were also no differences between students and school staff.

**Discussion:**

The results made it possible to evaluate the extent of SARS-CoV-2 transmission in the school community through immunity developed against the virus, in addition to providing information about COVID-19 symptoms in children, adolescents, and adults.

## 1 Introduction

On March 11, 2020, when the World Health Organization (WHO) declared the coronavirus disease 2019 (COVID-19) outbreak a global pandemic (1), several measures were taken, including the closure of schools for an indefinite period to diminish the spread of the virus (2). The decision impacted the lives of children and adolescents, affecting their educational performance and their physical, social, and mental well-being due to the loss of social contacts and school lunches (2–10).

Although data on the transmission of severe acute respiratory syndrome coronavirus 2 (SARS-CoV-2), number of cases, and deaths due to COVID-19 among children and adolescents are limited, there is evidence that reducing social contact among school-aged children during flu outbreaks decreases the transmission of the virus (3, 4). Some studies have shown a low prevalence of COVID-19 in children and adolescents under the age of 18 when compared with that in adults (5–7). The significant difference in the number of cases by age could be due to children being frequently asymptomatic, with mild or moderate illness and a low percentage of hospitalization, leading to a low demand for tests and the consequent underreporting of cases (5–10). The prevalence of confirmed COVID-19 cases in the pediatric population increased significantly in 2022 during the Omicron outbreak (9). This variant was more contagious than the earlier variants, with a higher viral binding affinity to the host cell receptor and immune evasion ability. However, a significantly lower risk of severe clinical outcomes has been observed in different pediatric age groups (9, 11).

Although uncommon, children could have two long-term consequences of SARS-CoV-2 infection, i.e., multisystem inflammatory syndrome (MIS-C) and “long COVID or post-acute sequelae of COVID-19” (PASC), which have severe clinical manifestations, including inflammation of parts of the body and the persistence, development, and oscillation of the signs and symptoms (8, 12).

In Brazil, after the reopening of schools in the second half of 2021, it became important to evaluate both asymptomatic and symptomatic SARS-CoV-2 infection in students, academic staff, and other school employees, as well as the record of previous COVID-19 disease and immunity against SARS-CoV-2. The data obtained can guide school managers in the implementation of measures to reduce the transmission of the virus and the possible risks of the disease (13–15).

Therefore, the present study aimed to assess the seroprevalence of the immunoglobulin G (IgG) anti-SARS-CoV-2 antibodies of the school community in the municipality of Campo Grande, state of Mato Grosso do Sul, from October 2021 to November 2022, and to perform a retrospective evaluation of the symptoms and their association with seropositivity.

## 2 Materials and methods

### 2.1 Study design and participants

**Figure 1:**
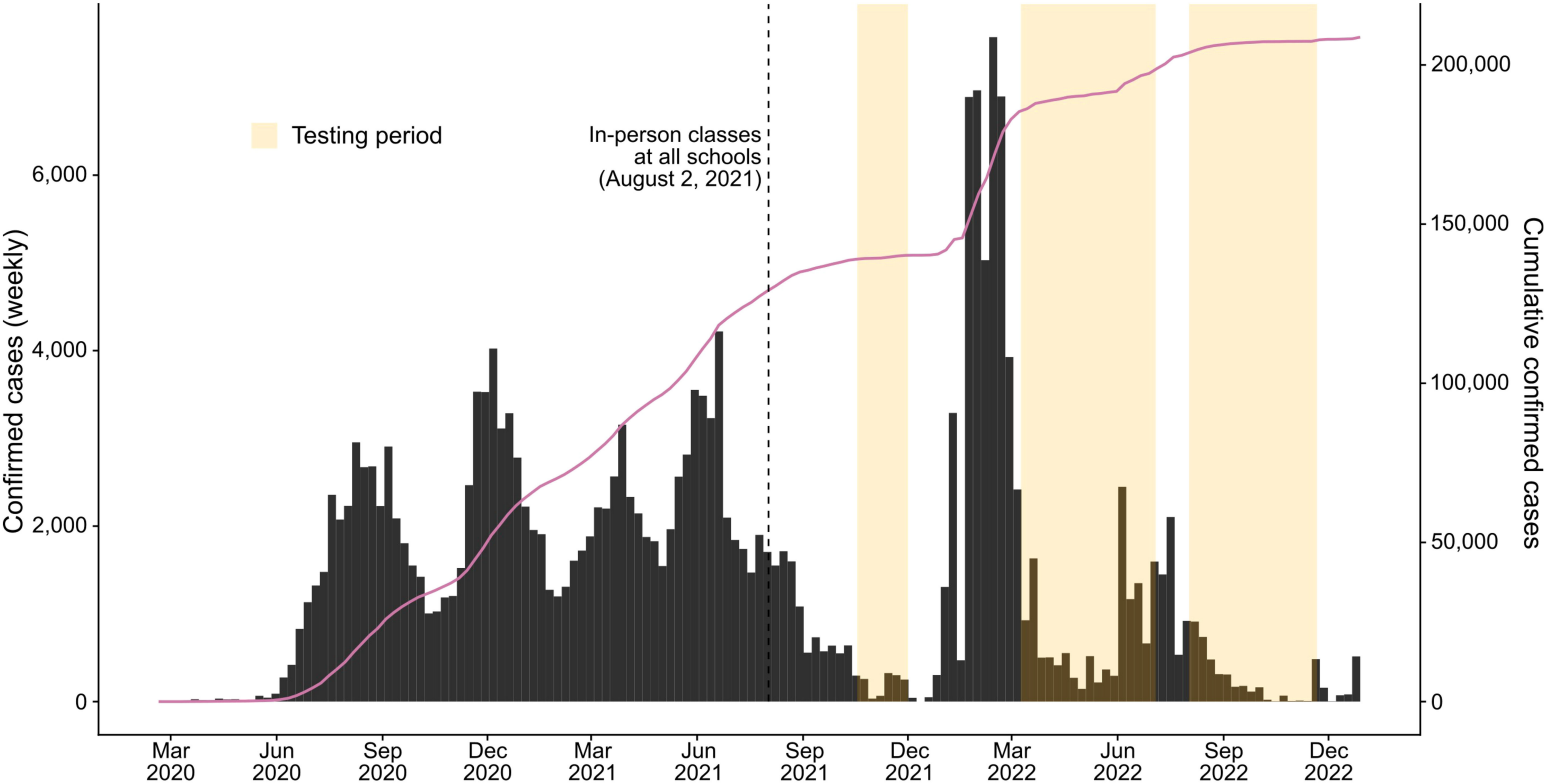
Weekly confirmed coronavirus disease 2019 (COVID-19) cases (*bars*) and cumulative cases (*solid line*) in the municipality of Campo Grande, Brazil, from 2020 to 2022. The *dashed line* shows the date on which all the schools had in-person classes. *Orange shading* denotes the testing periods for each survey. Data on confirmed cases for the municipality were obtained from https://covid.saude.gov.br/.

This is a cross-sectional serological survey performed in public and private schools in the urban areas of the municipality of Campo Grande, state of Mato Grosso do Sul, Brazil, from October 18, 2021 to November 21, 2022. The study was divided by school semester, with a total of three seroprevalence surveys: period 1, from October 18 to December 1, 2021, soon after the return of face-to-face classes; period 2, from March 9 to July 4, 2022; and period 3, from August 2 to November 21, 2022 (**Figure 1**). A total of 20 schools participated in the testing: 13 state schools, 5 municipal schools, and 2 private schools. Students aged between 6 and 17 years and professionals from school institutions, regardless of their roles (e.g., administrative, educational, or food preparation, among others) were considered eligible for enrolment. The present study is part of a larger and long-term research project, which is in accordance with the authorization of the Research Ethics Committee of Fundação Oswaldo Cruz (FIOCRUZ) of Brasília (CAAE: 47905721.9.0000.8027). For further details about the research project, see **Supplementary Data 1**.

### 2.2 Procedure

For each test period, eligible participants were contacted with the help of the administrators of each school through visits to advertise the project using posters and cell phone messages. A link was provided for the online registration of school members who agreed to fill out the Free and Informed Consent Form for participation in the research. Participants registered their personal information (e.g., age, sex, school name, school level, and period of activity) and answered a pandemic situation questionnaire regarding symptoms suggestive of COVID-19 from March 1, 2020 (e.g., fever, coryza, headache, sore throat, diarrhea, dyspnea, anosmia, or dysgeusia), self-reported previous positive tests for SARS-CoV-2, and vaccination for COVID-19. The registration of students aged 6–17 years was carried out by parents or guardians, who filled out all the information required in the questionnaire. After indicating their agreement in the Free and Informed Consent Form, the participants received an automatic copy in the email registered for contact. Participants who had difficulty completing the online registration were assisted by a team member, either in person or by phone. However, even with the consent of parents or guardians, the students received detailed information about the research and also signed the Free and Informed Assent Form, agreeing to participate in the study. A copy of the Free and Informed Assent Form was provided to be delivered to the respective parents or guardians (see **Supplementary Data 1** for more detail). In each period, the schools selected to develop the research project were visited every 2 weeks to test as many individuals as possible. Eligible participants were invited for testing in any of the three aforementioned periods. Those who accepted were submitted to antibody evaluation only once per sampling period. All enrolled students and professionals who were present at the time of the visit were tested following the manufacturer’s protocol (described in the next section), without repeating the test in the same period on those who were tested in the other visits. The study data were collected and managed using REDCap electronic data capture tools hosted at the Fundação Oswaldo Cruz of Mato Grosso do Sul—FIOCRUZ MS (16, 17).

### 2.3 Serological testing

Anti-SARS-CoV-2 antibodies were assessed using the rapid serological test TR DPP^®^ COVID-19 IgM/IgG produced by the Instituto de Tecnologia em Imunobiológicos (Bio-Manguinhos, FIOCRUZ, Rio de Janeiro, Brazil). The test uses anti-nucleocapsid (anti-N) antibodies to identify induced immunity, which are produced after natural infection and not after vaccination with spike-based vaccines (18). Testing involved a dual-path lateral flow immunochromatographic test that allows the simultaneous differentiation of the IgM and IgG antibodies from the same sample in two independent reactions. The qualitative detection of the antibodies was performed using a microreader that eliminated reading subjectivity and the possibility of human error. In the presence of specific antibodies, binding occurs with the conjugate and a pink line is produced, the intensity of which can be detected and quantified. The antibody level results were considered positive when the microreader reported ≥30 and negative when the value reported was <30. The assay had reported sensitivities of 79% (95% CI = 70.9–86.8) for IgM and 95% (95% CI = 88.8–97.9) for IgG, with specificities of 98% (95% CI = 95.8–99) and 97% (95% CI = 94–98) for IgM and IgG, respectively. Following the manufacturer‘s protocol, whole blood was collected from each participant using fingerstick capillary blood sampling.

### 2.4 Statistical analysis

Assuming a baseline seroprevalence of 3.1% (19), a sample size of 685 is the minimum needed to estimate the seroprevalence with a precision of ±1.5% at the 95% confidence level and with 25% of loss. The sample size was calculated using the ScalaR SP (20) in R software (21). The characteristics of the study participants were described by summarizing the demographics and clinical history of each using absolute frequencies and percentages. Symptoms suggestive of COVID-19 were presented descriptively, with absolute values separated by sampling period and serological test results. In addition, Pearson’s chi-squared test and Pearson’s residuals were used to examine the independence between responses on the symptoms suggestive of COVID-19 and previous positive tests for SARS-CoV-2. A significant result would indicate that the answers were possibly biased by participants with a previous positive test, not allowing associations to be made between the results of the serological test and the symptoms reported by the participants. These analyses were conducted using the R package “vcd“ (22–24).

Bayesian multilevel regression with post-stratification was used to obtain the seroprevalence estimates and the 95% CIs using the R package “rjags“ (25). However, only the detection of the IgG antibody was considered a positive result in the seroprevalence estimates due to the low number of positive results obtained for IgM and the low sensitivity of the test. Furthermore, the manufacturer used the same range of days to assess IgM and IgG detection, with the latter showing better performance. The model for each testing period included sex as a fixed effect and school level and school type (i.e., municipal, state, or private) as random effects. To generate population-representative seroprevalence estimates, these were weighted for sex, school level, and school type based on the 2021 and 2022 Brazilian Basic Education Census (26, 27). School level was chosen over age because of the better description of the census for these categories and the fact that participants tended to fill out information on school level better than age. As the survey was a non-random sample of the school community, post-stratification weights allowed adjustments for the total population size of the variables considered. Adjustment was done for test performance of IgG sensitivity and specificity. The model, weighting procedures, and the definitions of priors have been described in detail elsewhere (28, 29). Four chains of 10,000 iterations, each with 5,000 warm-up iterations, were used in the analyses. Convergence of the Markov chain Monte Carlo (MCMC) chains was assessed visually by trace, density, and running mean plots using the R package “mcmcplots“ (30) and with the Gelman and Rubin‘s convergence diagnostic (31, 32) using the R package “coda“ (33) (**Supplementary Figures S1–S12**). All statistical analyses were conducted using R software v4.3.0.

## 3 Results

A total of 1,234 eligible participants initially agreed to participate in the research in testing period 1; however, 489 individuals (39.6%) were unavailable at the time of the survey. Six individuals were excluded because they were registered as students, but reported being over the age of 17 years, leaving 739 participants. In the second period, 807 eligible subjects agreed to participate in the study, but 110 (13.6%) were unavailable at the time and two were outside the authorized criteria (students over 17 years of age), leaving 695 participants. Finally, 827 eligible participants agreed to participate in testing period 3, but only 712 were enrolled because 112 (13.5%) were not available at the time and three were outside the authorized criteria (students over the age of 17 years) (**Table 1**; **Supplementary Data 2–4**).

**Table 1:** Characteristics of the participants.

		Period 1	Period 2	Period 3
Total		739	695	712
Sex	Women	512 (69.3%)	489 (70.4%)	514 (72.2%)
	Men	227 (30.7%)	206 (29.6%)	198 (27.8%)
Age (years)	6–10	111 (15.0%)	89 (12.8%)	69 (9.7%)
	11–14	200 (27.1%)	163 (23.5%)	152 (21.3%)
	15–17	139 (18.8%)	85 (12.2%)	86 (12.1%)
	≥18	275 (37.2%)	354 (50.9%)	401 (56.3%)
	No data	14 (1.9%)	4 (0.6%)	4 (0.6%)
School level/staff	Primary	132 (17.9%)	85 (12.2%)	75 (10.5%)
	Lower secondary	210 (28.4%)	162 (23.3%)	151 (21.2%)
	Upper secondary	117 (15.8%)	88 (12.7%)	80 (11.2%)
	Staff	280 (37.9%)	360 (51.8%)	406 (57.0%)
School type	Municipal	263 (35.6%)	226 (32.5%)	225 (31.6%)
	Private	110 (14.9%)	82 (11.8%)	78 (11.0%)
	State	366 (49.5%)	387 (55.7%)	409 (57.4%)
Self-reported previous positive test	Yes	153 (20.7%)	193 (27.8%)	219 (30.8%)
	No	523 (70.8%)	484 (69.6%)	480 (67.4%)
	No data	63 (8.5%)	18 (2.6%)	13 (1.8%)
Vaccinated	Yes	521 (57.0%)	617 (88.8%)	647 (90.9%)
	No	158 (21.4%)	59 (8.5%)	51 (7.2%)
	No data	160 (21.6%)	19 (2.7%)	14 (2.0%)

Values indicate the number of participants tested per period in each category.

Across the testing periods, most of the participants were women (≥69%), with a mean age of 24 years (range, 6–72 years) in testing period 1, 28 years (range, 6–70 years) in period 2, and 30 years (range, 6–70 years) in period 3 (**Table 1**). Although the average number of participants per school remained constant (**Table 2**), there was a decrease in student participation and an increase in school staff participation during each testing period (**Tables 1**, **2**). In addition, a higher proportion of participants reported having previously tested positive and were vaccinated in each period (**Table 1**).

**Table 2:** Participation rates for each testing period, shown as the median number of participants per school (minimum–maximum; number of schools).

		Period 1	Period 2	Period 3
Total		37 (7–132; 20)	35 (4–75; 20)	36 (6–76; 20)
Sex	Women	26 (4–94; 20)	24 (4–50; 20)	26 (5–52; 20)
	Men	11 (1–38; 20)	11 (3–25; 18)	10 (1–24; 19)
School level/staff	Primary	13 (1–43; 10)	7 (1–17; 13)	6 (1–17; 12)
	Lower secondary	12 (1–36; 17)	9 (2–22; 19)	8 (1–20; 19)
	Upper secondary	8 (1–18; 14)	7 (1–19; 13)	7 (2–18; 12)
	Staff	14 (1–53; 20)	19 (1–39; 19)	20 (3–44; 20)
School type	Municipal	53 (14–132; 5)	45 (23–64; 5)	45 (20–65; 5)
	Private	55 (53–57; 2)	41 (27–55; 2)	39 (26–52; 2)
	State	28 (7–83; 13)	30 (4–75; 13)	31 (6–76; 13)

During the first testing period, 162 out of 739 participants were seropositive for anti-SARS-CoV-2 antibodies (IgM = 8, IgM and IgG = 19, IgG = 135), with 154 seropositive for IgG antibodies. In the second period, 251 out of 695 participants were seropositive (IgM = 6, IgM and IgG = 113, IgG = 132), with 245 having IgG antibodies. In the third period, 393 out of 712 participants were seropositive (IgM = 4, IgM and IgG = 163, IgG = 226), with 389 participants having IgG antibodies. Less than half of the participants who reported a positive test in the questionnaire before being evaluated in period 1 were seropositive; however, this percentage increased in the second and third periods, which included those who did not test positive. The Bayesian population-weighted and test-adjusted seroprevalence rates were 19.6% (95% CI = 15.2–24.3) for the first period, 34.6% (95% CI = 29.4–40.0) for the second period, and 56.7% (95% CI = 51.1–62.7) for the third period (**Table 3**). Despite the significant increase in the estimated seroprevalence in each period, there was little variation within each evaluated period across sex, school level, and school type (**Table 3**).

**Table 3:** Estimated seroprevalence of anti-severe acute respiratory syndrome coronavirus 2 (SARS-CoV-2) antibodies.

		Period 1		Period 2		Period 3	
		Sp (IgG)	ESp (95% CI)^a^	Sp (IgG)	ESp (95% CI)^a^	Sp (IgG)	ESp (95% CI)^a^
Total		162 (154)	19.6% (15.2–24.3)	251 (245)	34.6% (29.4–40.0)	393 (389)	56.7% (51.1–62.7)
Sex	Women	114 (108)	19.3% (14.2–24.8)	189 (183)	34.0% (27.6–40.7)	288 (284)	54.6% (47.5–61.7)
	Men	48 (46)	19.1% (13.3–25.4)	62 (62)	34.8% (27.1–42.8)	105 (105)	60.4% (52.0–69.0)
Children according to school level and staff	Primary	30 (30)	19.7% (14.4–25.6)	30 (29)	34.5% (27.4–41.9)	41 (41)	54.6% (46.0–62.3)
	Lower secondary	40 (38)	20.1% (14.1–27.6)	63 (62)	32.9% (24.1–41.4)	88 (88)	56.5% (48.1–65.1)
	Upper secondary	24 (24)	19.4% (14.1–25.1)	29 (29)	38.9% (31.9–47.2)	41 (41)	58.6% (51.9–66.2)
	Staff	68 (62)	17.3% (10.1–23.6)	129 (125)	30.7% (21.2–39.2)	223 (219)	55.7% (46.9–63.8)
School type	Municipal	69 (66)	17.9% (12.7–23.1)	85 (81)	32.6% (26.1–38.9)	114 (113)	57.0% (50.5–63.8)
	Private	21 (21)	20.7% (14.6–28.2)	31 (30)	34.8% (27.7–43.2)	49 (49)	54.7% (46.4–62.7)
	State	72 (67)	17.7% (12.6–22.8)	135 (134)	34.8% (28.5–41.6)	230 (227)	58.3% (51.4–65.4)

Sp, total number of seropositives for the anti-SARS-CoV-2 antibodies; IgG, total number of seropositives for only IgG or IgG or both (IgM and IgG); ESp, estimated seroprevalence; CI, confidence interval.

a Weighted for school type, school level, and sex and adjusted for test sensitivity and specificity for the IgG antibody.

**Figure 2:**
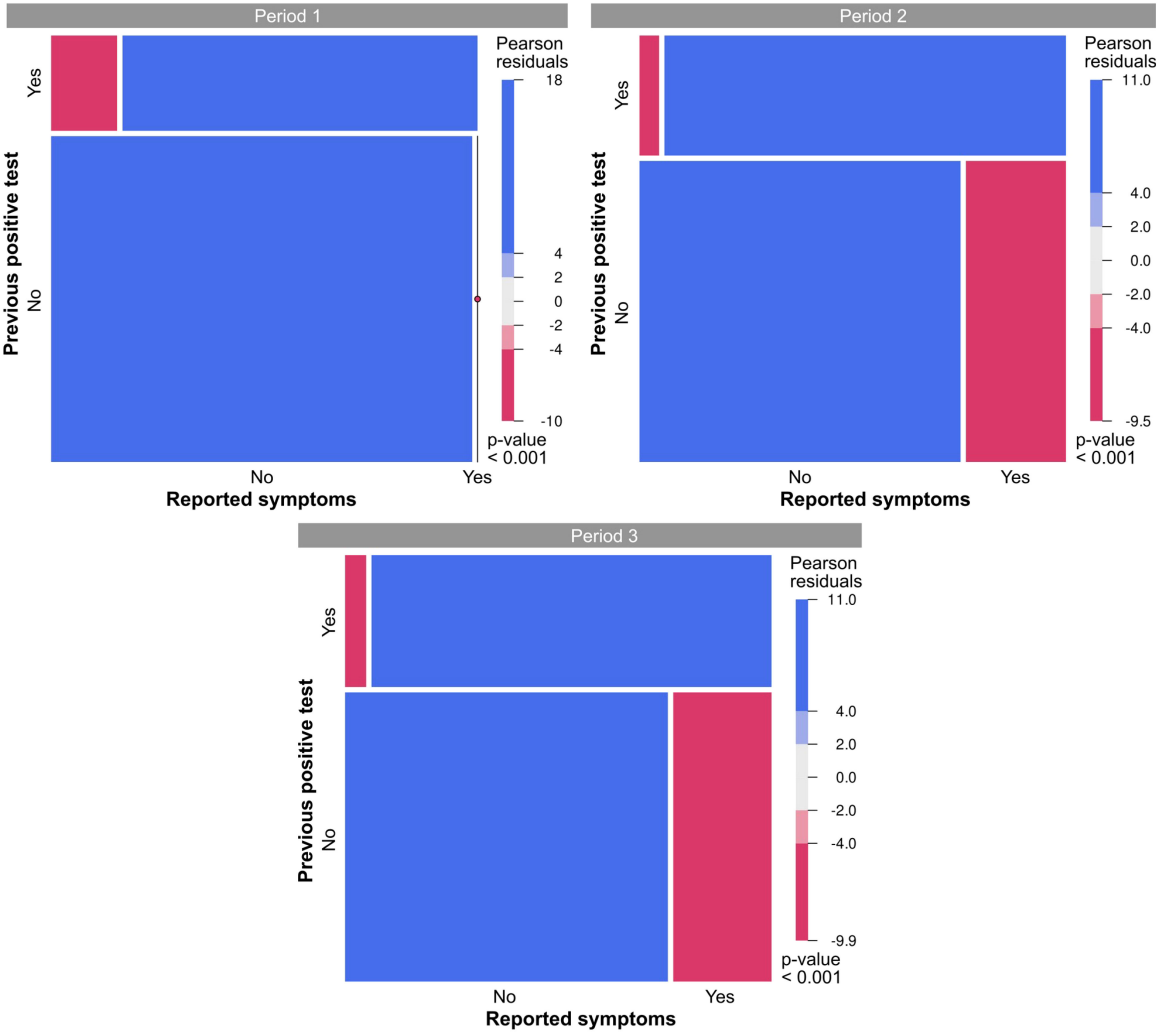
Mosaic plots illustrating the distribution of responses regarding symptoms suggestive of coronavirus disease 2019 (COVID-19) and previous positive tests for severe acute respiratory syndrome coronavirus 2 (SARS-CoV-2). The *size of the tiles* corresponds to the number of cases that fall within each category. *Colors* indicate deviations from the null hypothesis of independence. *Blue*, category is overrepresented; *red*, category is underrepresented.

**Figure 3:**
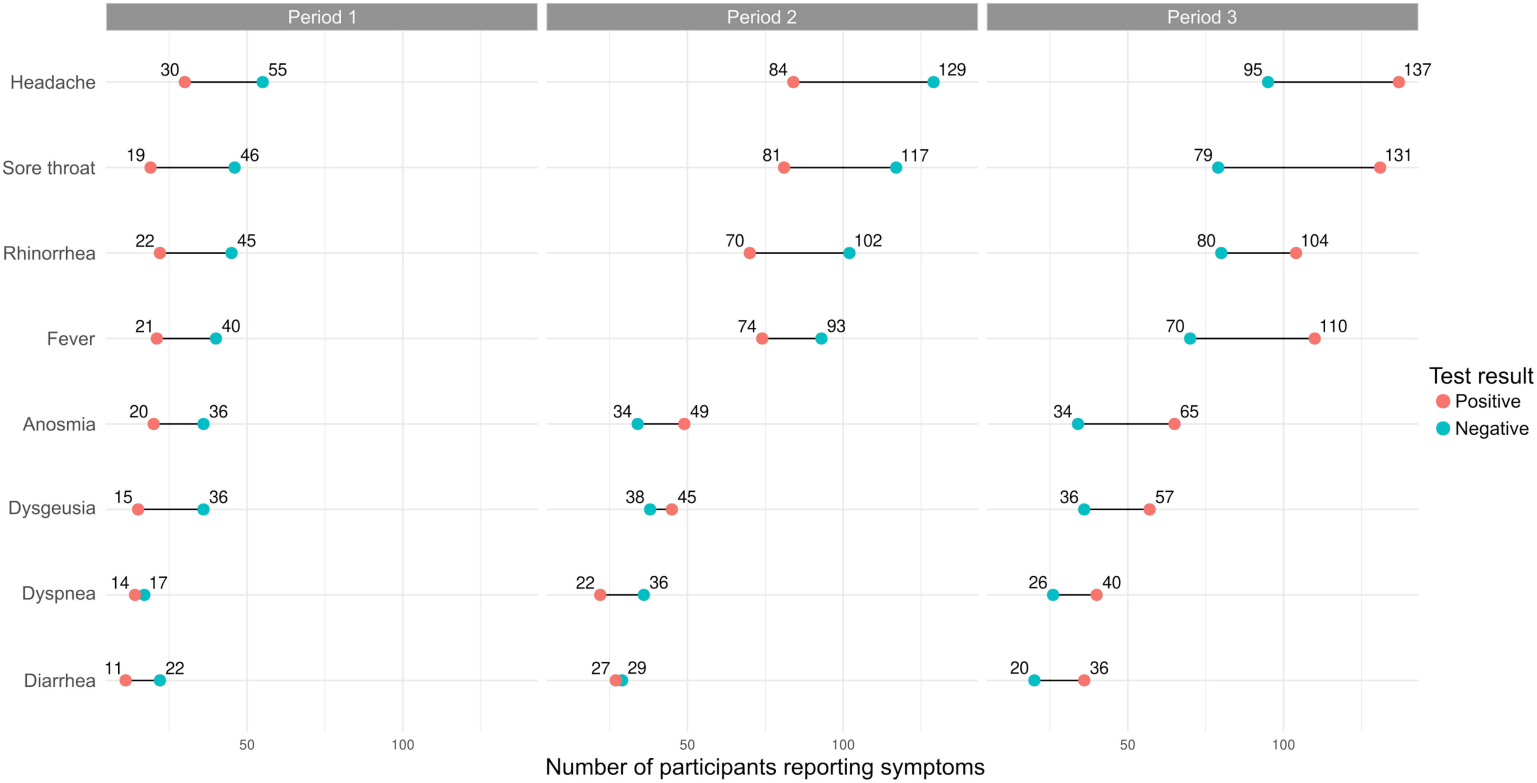
Frequency of self-reported symptoms in seropositive and seronegative participants for each testing period.

Among all participants, those who mentioned experiencing symptoms suggestive of COVID-19 since March 1, 2020, were primarily those who reported having previously tested positive, suggesting a possible symptom-reporting bias (**Figure 2**). In general, the most frequently listed symptoms during the three testing periods were headache, sore throat, rhinorrhea, and fever. Although anosmia and dysgeusia have been widely mentioned, especially among seropositive cases, these symptoms were not as commonly reported during testing periods 2 and 3 (**Figure 3**).

## 4 Discussion

This research represents the first serological survey of antibodies against SARS-CoV-2 conducted in school institutions in the city of Campo Grande, Mato Grosso do Sul, Brazil. Comprehension of the role of children and adolescents in the transmission of the new coronavirus has generated wide scientific discussions since the beginning of the pandemic. In 2021, when schools returned to in-person classes, it was a topic of great relevance to evaluate the circulation of SARS-CoV-2 among students and staff, as well as the symptoms reported when developing the disease (34–38).

According to the epidemiological bulletin of the state of Mato Grosso do Sul, from January to December 2021, the number of positive cases for COVID-19 ranged between 161,371 and 380,873, with a higher prevalence of the variant of concern (VOC), P1.1.* (Gamma), detected for the first time in the state of Amazonas (Brazil) in January 2021, which had a higher transmissibility than preexisting lineages (39). From August to December, the number of new cases of COVID-19 decreased from 12,619 in August to 1,603 in December. In August and September, the Gamma variant was still detected in positive samples. From September to December, the variant B.1.617.2+AY.* (Delta) began to circulate in the state, a VOC detected in the country for the first time since June 2021 (40–42). In Campo Grande, the same trend was observed for the number of cases (**Figure 1**). However, unlike the state, from August to December, the number of new cases also increased (from 134,588 to 140,120), although the increase was not as high from one month to the next (41).

The overall seroprevalence estimated in the study, from October to December 1, was 19.6% (95% CI = 15.2–24.3) (**Table 3**). Prevention and protection measures, such as the use of masks and alcohol gel, frequent cleaning of contact surfaces, hand hygiene, and vaccination of individuals over 12 years of age (which started in January 2021) could have contributed to the low percentage of infected individuals in the population studied (43, 44). In 2020, serological surveys were conducted in children and adults in the school community in other states of Brazil, specifically in the city of São Paulo, state of São Paulo (45), and in Fortaleza, state of Ceará (46). In São Paulo, a seroprevalence of 16.6% for SARS-CoV-2 antibodies was found in schoolchildren. However, in municipal (18.5%) and state (16.2%) schools from the public system, the detection of antibodies was higher than that in private schools (11.7%). In Fortaleza, the seroprevalence rates were 25.3% among children, 29.2% among adolescents, and 20.9% among adults. However, no significant differences were found in the seroprevalence rates between the sampled groups (46).

In the study developed in the city of Campo Grande (state of Mato Grosso do Sul), during the second and third testing periods, the estimated seroprevalence increased to 34.6% and 56.7%, respectively, possibly a consequence of the outbreak of cases associated with the variant BA.1.* (Omicron), which was a more contagious VOC associated with less severe COVID-19 infection compared to that caused by the Delta variant (47). Omicron presented greater humoral immune escape, thereby reducing the effectiveness of vaccines (48, 49). Despite this increase in transmissibility, the indicators of clinical severity were higher for VOC Gamma, a variant prevalent in Mato Grosso do Sul from March to August 2021 (50). In addition, with the increase in the vaccination rate of the population, state and municipal government institutions in Brazil ceased to require the mandatory use of masks in public places, private establishments accessible to the public, and in public transportation.

As in other studies (50, 51), no statistical difference was found in seropositivity by sex, although women represented approximately 70% of the population tested (**Table 3**). In addition, when estimating the seroprevalence in the different age groups according to the school level or the school type (i.e., municipal, state, or private), no statistical differences within each group were found (**Table 3**). At the beginning of the pandemic, some studies have reported a lower susceptibility of children to SARS-CoV-2 infection than adults (52). According to Chou et al. (53), in children and adolescents, there is less expression of the angiotensin-converting enzyme 2 (ACE2), which is present in abundance on the surface of endothelium cells in the kidneys, lungs, and other organs in adults and functions as a receptor for the spike protein from SARS-CoV-2, facilitating its entry into the host cell.

The clinical manifestations of individuals infected with SARS-CoV-2 are diverse. In the present study, the symptoms most frequently reported by participants in the screening questionnaire were headache, sore throat, runny nose, and fever, consistent with other studies (45, 54, 55). However, memory bias undoubtedly influenced the self-reported symptoms (**Figure 2**), particularly those reported during the first testing period (**Figure 3**). With the study already underway and with the increase in cases from the second testing period onwards (**Figure 1**; **Table 3**), participants were possibly able to report symptoms more accurately. This was evidenced by the change in the frequency of symptoms among seropositive and seronegative participants observed over the first and third periods, with symptoms more frequently reported by those who were seropositive in the third period (**Figure 3**). However, as this was a retrospective survey of symptoms based on self-report and recall, the extent to which these symptoms may be associated with COVID-19 is uncertain, and any generalizations could be misleading (56, 57). There have been no reports of comorbidities between children and adolescents; therefore, they are less likely to develop severe disease (58).

This study has some limitations. Firstly, the number of participants was reduced, which made it difficult to compare subgroups (**Tables 1**, **2**). Secondly, there were manifestations of mild or asymptomatic infection in some individuals, and in these cases, the production of antibodies may have been at low levels and undetectable by the test used. Finally, the rapid serological test allowed us to determine whether the participant had contact with SARS-CoV-2 and to estimate the level of exposure of the school population. IgM antibodies could be detected in sample blood at an early stage of the infection, establishing a short-term response; later, IgG is produced and persists for at least several months in most individuals. The precise duration of IgG antibodies in the body is unknown, and it is difficult to identify when the infection occurred (59). At the beginning of the survey, when participants registered on the REDCap platform, they mentioned the date on which they had COVID-19 as confirmed by the molecular test. However, if there was an infection before or after completing the questionnaire, either asymptomatic or with mild symptoms, which was not identified through any laboratory test, it will not be possible to differentiate with the use of the serological test.

The number of participants remained constant during the three periods of the study; however, there was a change in the proportions of children, adolescents, and adults participating, verifying, during the research, an increase in the number of professionals and a decrease in the number of students (**Tables 1**, **2**). Ahmed et al. (60) also observed a drop in the participation of children throughout the study. Ulyte et al. (61) mentioned that the anxiety generated in performing the rapid test, when needing to pierce the finger, could probably be the reason for some of the volunteers giving up on continuing to participate in the study. In this sense, the test results should be interpreted with caution and should be used in conjunction with other information to analyze the epidemiology of the novel coronavirus in a specific locality or population and, thus, be able to propose strategies for the prevention and control of the transmission of the virus.

Although some individuals have reported discomfort during digital puncture, the availability of high-quality tests that allow the detection of antibodies against the new coronavirus constitutes a valuable tool for epidemiological surveillance and for understanding of the transmission of SARS-CoV-2 in different groups of age, sex, and demographics in the school community. The analysis of population immunity can serve as guidance for health managers and school institutions concerning the strategies that can be implemented for the prevention and control of respiratory virus transmission among students, teachers, and other professionals in the school environment. This information can be used to identify risk groups and adjust the following biosecurity protocols according to the needs of the school community.

Thus, this pioneering study in Mato Grosso do Sul highlights the importance of the continuous surveillance of seroprevalence against SARS-COV-2 to assess the extent of transmission in the school community and thus guide managers toward necessary prevention measures when an increase in seroprevalence or even the relaxation of measures is observed in the case of reduced seroprevalence.

## Data availability statement

The original contributions presented in the study are included in the article/**Supplementary Material**. Further inquiries can be directed to the corresponding authors.

## Ethics statement

The studies involving humans were approved by Research Ethics Committee of Fundação Oswaldo Cruz (FIOCRUZ) of Brasília (CAAE: 47905721.9.0000.8027). The studies were conducted in accordance with the local legislation and institutional requirements. Written informed consent for participation in this study was provided by the participants’ legal guardians/next of kin.

## Author contributions

ZCF: Writing – review & editing, Writing – original draft, Visualization, Supervision, Resources, Project administration, Methodology, Investigation, Conceptualization. CS: Writing – review & editing, Methodology, Investigation, Data curation. DMCA: Writing – review & editing, Validation, Supervision, Methodology, Investigation, Formal analysis, Data curation. CMS: Writing – review & editing, Project administration, Methodology, Investigation. JMT: Writing – review & editing, Methodology, Investigation. RR: Writing – review & editing, Methodology, Investigation. GEBM: Writing – review & editing, Methodology, Investigation. ECF: Writing – review & editing, Methodology, Investigation. ECFBS: Writing – review & editing, Supervision, Investigation. TFO: Writing – review & editing, Supervision, Resources. FMLM: Resources, Writing – review & editing, Supervision, Conceptualization. EFL: Writing – review & editing, Supervision, Software. LHFD: Writing – review & editing, Supervision, Project administration. GGCL: Writing – review & editing, Methodology, Investigation. MCSUZ: Writing – review & editing, Supervision, Project administration. CCMG: Writing – review & editing, Supervision, Project administration. JFG: Visualization, Writing – review & editing, Supervision, Resources, Project administration. RTP: Writing – review & editing, Visualization, Project administration.
