# Supplementary material for: Seroprevalence of antibodies against SARS-CoV-2 in the school community in Campo Grande, state of Mato Grosso do Sul, Brazil, October 2021–November 2022
Source: Front Immunol. 2024 Mar 26;15:1354786. doi: 10.3389/fimmu.2024.1354786 (PMC11002276; doi:10.3389/fimmu.2024.1354786)
Supplement: Supplementary file 1 [file DataSheet_1.pdf]

Supplementary Material

Supplementary Material on methods and results

Contents

Supplementary Data 1. About the project and the school context in the municipality during the COVID-19 pandemic........ 2

Supplementary Data 2. Dataset of the first testing period (from October 18 to December 1, 2021)... 5

Supplementary Data 3. Dataset of the second testing period (from March 9 to July 4, 2022)........... 6

Supplementary Data 4. Dataset of the third testing period (from August 2 to November 21, 2022)... 7

Supplementary Figure 1. Markov chain Monte Carlo (MCMC) trace plots for the model from the first test period (from October 18 to December 1, 2021)........ 8

Supplementary Figure 2. Markov chain Monte Carlo (MCMC) density plots for the model from the first testing period (from October 18 to December 1, 2021)........ 9

Supplementary Figure 3. Markov chain Monte Carlo (MCMC) running mean plots for the model from the first testing period (from October 18 to December 1, 2021)........ 10

Supplementary Figure 4. Plots of the evolution of Gelman and Rubin's shrink factor as the number of iterations increases for the model from the first testing period (from October 18 to December 1, 2021)........ 11

Supplementary Figure 5. Markov chain Monte Carlo (MCMC) trace plots for the model from the second testing period (from March 9 to July 4, 2022)........ 12

Supplementary Figure 6. Markov chain Monte Carlo (MCMC) density plots for the model from the second testing period (from March 9 to July 4, 2022)........ 13

Supplementary Figure 7. Markov chain Monte Carlo (MCMC) running mean plots for the model from the second testing period (from March 9 to July 4, 2022)........ 14

Supplementary Figure 8. Plots of the evolution of Gelman and Rubin's shrink factor as the number of iterations increases for the model from the second testing period (from March 9 to July 4, 2022).. 15

Supplementary Figure 9. Markov chain Monte Carlo (MCMC) trace plots for the model from the third testing period (from August 2 to November 21, 2022)........ 16

Supplementary Figure 10. Markov chain Monte Carlo (MCMC) density plots for the model from the third testing period (from August 2 to November 21, 2022)........ 17

Supplementary Figure 11. Markov chain Monte Carlo (MCMC) running mean plots for the model from the third testing period (from August 2 to November 21, 2022)........ 18

Supplementary Figure 12. Plots of the evolution of Gelman and Rubin's shrink factor as the number of iterations increases for the model from the third testing period (from August 2 to November 21, 2022)........ 19

Supplementary Data 1. About the project and the school context in the municipality during the COVID-19 pandemic.

This study is part of a larger and long-term research project entitled "The return to school and the transmission of respiratory viruses in schools in Campo Grande, Brazil". The main goals of this project are to investigate the circulation of SARS-CoV-2 and the social and psychological impact of COVID-19 pandemic in public and private schools in the urban area of the municipality of Campo Grande, state of Mato Grosso do Sul, Brazil. Sociodemographic information was collected, and molecular and serological tests were performed on students (aged 6–17 years), teachers, and other employees of educational institutions. In addition, it is also intended to evaluate the circulation of other respiratory viruses, including Influenza A and B, respiratory syncytial virus, metapneumovirus, adenovirus, and rhinovirus, in participants who present symptoms and have an undetectable result for SARS-CoV-2. The research project is developed by Oswaldo Cruz Foundation of Mato Grosso do Sul (FIOCRUZ MS), in partnership with the Immunobiological Technology Institute (Bio-Manguinhos - FIOCRUZ), the Central Public Health Laboratory of the state of Mato Grosso do Sul (LACEN-MS), the State Department of Health and the State Department of Education of the state of Mato Grosso do Sul (SES-MS and SED-MS), the Municipal Department of Education of Campo Grande (SEMED), the Federal University of Mato Grosso do Sul (UFMS), and the Catholic University of Don Bosco (UCDB). The project started on October 18, 2021 and is conducted in accordance with the authorization of the Research Ethics Committee of FIOCRUZ Brasília (CAAE: 47905721.9.0000.8027).

In-person classes were suspended on March 17, 2020, in the municipality of Campo Grande, Mato Grosso do Sul, Brazil. The in-person return to school took place on July 26, 2021, for municipal schools and on August 2, 2021, for state schools. On the other hand, private schools returned to in-person classes on April 5, 2021, but with a hybrid option for the rotation of students or families who opt for the online system. Biosafety standards were established to try to prevent the spread of SARS-CoV-2 upon the return to in-person classes by 2021. At the entrance to the units, everyone's temperature was measured and the use of a mask was mandatory. In addition, hand washing and other care for personal hygiene and the environment were common guidelines on school premises. Meanwhile, to ensure the safe return of students and professionals, a schedule of students was established so that everyone could attend in-person classes and avoid agglomerations. As a result, the classrooms had occupancies between 25% and 50% according to the size of the space to guarantee the necessary distance. The "Standard Operating Procedures" document (SOPs) was prepared by each school, taking into account the number of students and the size of the classrooms. In the staggered return, students were divided into groups of 30% to 50%, in the

amount necessary to ensure adequate distance in the classroom, with a weekly rotation. In this way, students had one-week in-person classes and remote classes in the following weeks. Students who were part of the risk group (with comorbidities) could return to in-person classes only with the presentation of a medical statement authorizing them, and parents had to sign a term of responsibility. Unlike 2021, the 2022 school year returned to full in-person classes. Also, in March 2022, the governor of the state of Mato Grosso do Sul published a decree ending the mandatory use of personal protective masks in any location (both closed and open places). The decree came into effect on March 10, 2022, and marked the end of preventive measures against the transmission of SARS-CoV-2 in the state.

According to the Brazilian Basic Education Census, in 2021, Campo Grande had 418 schools in the urban area with students within the age range evaluated in the project, with a total of 144,123 student registrations at these ages, according to the Instituto Nacional de Estudos e Pesquisas Educacionais Anísio Teixeira- INEP (INEP, 2022). By 2022, there were 461 schools and 145,119 students within the study age range (INEP, 2023). The organizational structure and responsibilities of education in Brazil are defined as follows: states must ensure elementary education (primary and lower secondary school) and offer, as a priority, upper secondary education to all who demand it; municipalities must offer early childhood education in day care centers and preschools, and, as a priority, elementary education (primary and lower secondary school); and private institutions can contemplate any stage of education. Despite the authorizations to conduct the project, the schools had the autonomy to decide whether to participate, as well as withdraw from participation at any time. Public (municipal and state schools) and private schools were contacted and invited to participate in the project. However, schools that had both primary and secondary education were prioritized to facilitate sampling of all educational stages targeted by the study, without having to sample a large number of schools. Approximately 250 schools met this requirement, of which 114 schools were contacted. In total, 23 schools agreed to participate, 14 of which were state schools, five municipal schools and four private schools. However, three schools that participated in 2021 decided not to participate by 2022. Interestingly, three schools that did not participate in 2021 decided to participate in 2022. Thus, the number of schools remained at 20 in 2021 and 2022.

People interested in participating in the survey received a link to register for the project using a computer or mobile device, in which the participants had to accept the consent form, introduce their personal information, and answer demographic and pandemic situation questionnaires. However, participation was conditioned by the acceptance of the Free and Informed Consent Form and the presentation of personal information. The same obligation was not attributed

to the questionnaires, due to the difficulty that some of the participants had in remembering some information. The non-requirement to fill in the demographic and pandemic questionnaires for participation was defined to increase the number of enrolled participants. To address this situation as the project progressed, registered participants were contacted by phone to request and help them fill in all the responses. However, it was not always possible to meet the participants, even by telephone, especially when it came to the legal representatives of the students. This was certainly one of the greatest difficulties of the project, convincing people to participate and obtaining the necessary information to carry out the project. Thus, missing data were in the variables: age, self-reported previous positive test for SARS-CoV-2, and COVID-19 vaccination. The values and percentages of missing data can be seen in Table 1 of the manuscript.

Reference

1. INEP – Instituto Nacional de Estudos e Pesquisas Educacionais Anísio Teixeira. Microdados do Censo da Educação Básica 2021. (2022) https://www.gov.br/inep/pt-br/acesso-a-informacao/dados-abertos/microdados/censo-escolar [Accessed September 6, 2023]
2. INEP – Instituto Nacional de Estudos e Pesquisas Educacionais Anísio Teixeira – INEP. Microdados do Censo da Educação Básica 2022. (2023) https://www.gov.br/inep/pt-br/acesso-a-informacao/dados-abertos/microdados/censo-escolar [Accessed September 6, 2023]

Supplementary Data 2. Dataset of the first testing period (from October 18 to December 1, 2021). It shows the number of both tests performed and seropositive results by school type, education degree and sex. The population size was obtained from the 2021 Basic Education Census. Lw Sec, Lower Secondary. Up Sec, Upper Secondary.

School Type	Education degree	Sex	Tested	Positives	Population	Population weight
State	Primary	Female	10	3	1448	0.00889494990447758
		Male	13	1	1542	0.00947238449772405
	Lw Sec	Female	67	12	6293	0.0386574031414899
		Male	44	5	6602	0.0405555657937576
	Up Sec	Female	62	12	14602	0.0896989354317552
		Male	34	8	14567	0.089483933189589
	Staff	Female	115	23	3256	0.020001351442665
		Male	21	3	1023	0.00628420839245895
Municipal	Primary	Female	45	12	22689	0.139376739214566
		Male	38	9	24151	0.14835769001591
	Lw Sec	Female	32	9	13733	0.0843607369048277
		Male	20	5	14406	0.0884949228756243
	Up Sec	Female	0	0	0	0
		Male	0	0	0	0
	Staff	Female	118	26	4793	0.0294430213343653
		Male	10	5	1506	0.00925123933435306
Private	Primary	Female	15	3	6327	0.0388662624624514
		Male	11	2	6734	0.0413664313927845
	Lw Sec	Female	21	3	4306	0.0264514187076522
		Male	26	4	4517	0.0277475750818544
	Up Sec	Female	13	2	2543	0.0156214486236785
		Male	8	2	2536	0.0155784481752453
	Staff	Female	14	3	3968	0.0243751113404468
		Male	2	2	1247	0.00766022274232288

Supplementary Data 3. Dataset of the second testing period (from March 9 to July 4, 2022). It shows the number of both tests performed and seropositive results by school type, education degree and sex. The population size was obtained from the 2022 Basic Education Census. Lw Sec, Lower Secondary. Up Sec, Upper Secondary.

School Type	Education degree	Sex	Tested	Positives	Population	Population weight
State	Primary	Female	5	3	779	0.0047117030477884
		Male	9	2	830	0.00502017141163591
	Lw Sec	Female	58	26	6545	0.0395867733604302
		Male	33	8	6865	0.041522261133591
	Up Sec	Female	48	19	13373	0.0808852437202494
		Male	31	7	13341	0.0806916949429334
	Staff	Female	169	61	3560	0.0215323014764143
		Male	34	8	1119	0.00676815880677179
Municipal	Primary	Female	19	5	23050	0.139415603660491
		Male	33	12	24535	0.148397476607816
	Lw Sec	Female	32	13	13527	0.0818166972110831
		Male	18	8	14189	0.0858207375418096
	Up Sec	Female	0	0	0	0
		Male	0	0	0	0
	Staff	Female	107	34	7359	0.044510170383408
		Male	17	9	2312	0.013983899161087
Private	Primary	Female	7	3	6656	0.0402581456817453
		Male	12	4	7086	0.0428589573769302
	Lw Sec	Female	12	5	4524	0.0273629583930613
		Male	9	2	4746	0.0287057030356916
	Up Sec	Female	5	3	2697	0.0163125328881711
		Male	4	0	2691	0.0162762424924244
	Staff	Female	27	11	4222	0.0255363418071407
		Male	6	2	1327	0.00802622585932633

Supplementary Data 4. Dataset of the third testing period (from August 2 to November 21, 2022). It shows the number of both tests performed and seropositive results by school type, education degree and sex. The population size was obtained from the 2022 Basic Education Census. Lw Sec, Lower Secondary. Up Sec, Upper Secondary.

School Type	Education degree	Sex	Tested	Positives	Population	Population weight
State	Primary	Female	5	4	779	0.0047117030477884
		Male	7	6	830	0.00502017141163591
	Lw Sec	Female	57	36	6545	0.0395867733604302
		Male	26	11	6865	0.041522261133591
	Up Sec	Female	44	22	13373	0.0808852437202494
		Male	29	16	13341	0.0806916949429334
	Staff	Female	201	111	3560	0.0215323014764143
		Male	40	21	1119	0.00676815880677179
Municipal	Primary	Female	17	7	23050	0.139415603660491
		Male	30	14	24535	0.148397476607816
	Lw Sec	Female	29	15	13527	0.0818166972110831
		Male	17	9	14189	0.0858207375418096
	Up Sec	Female	0	0	0	0
		Male	0	0	0	0
	Staff	Female	112	56	7359	0.044510170383408
		Male	20	12	2312	0.013983899161087
Private	Primary	Female	6	4	6656	0.0402581456817453
		Male	10	6	7086	0.0428589573769302
	Lw Sec	Female	13	10	4524	0.0273629583930613
		Male	9	7	4746	0.0287057030356916
	Up Sec	Female	3	2	2697	0.0163125328881711
		Male	4	1	2691	0.0162762424924244
	Staff	Female	27	17	4222	0.0255363418071407
		Male	6	2	1327	0.00802622585932633

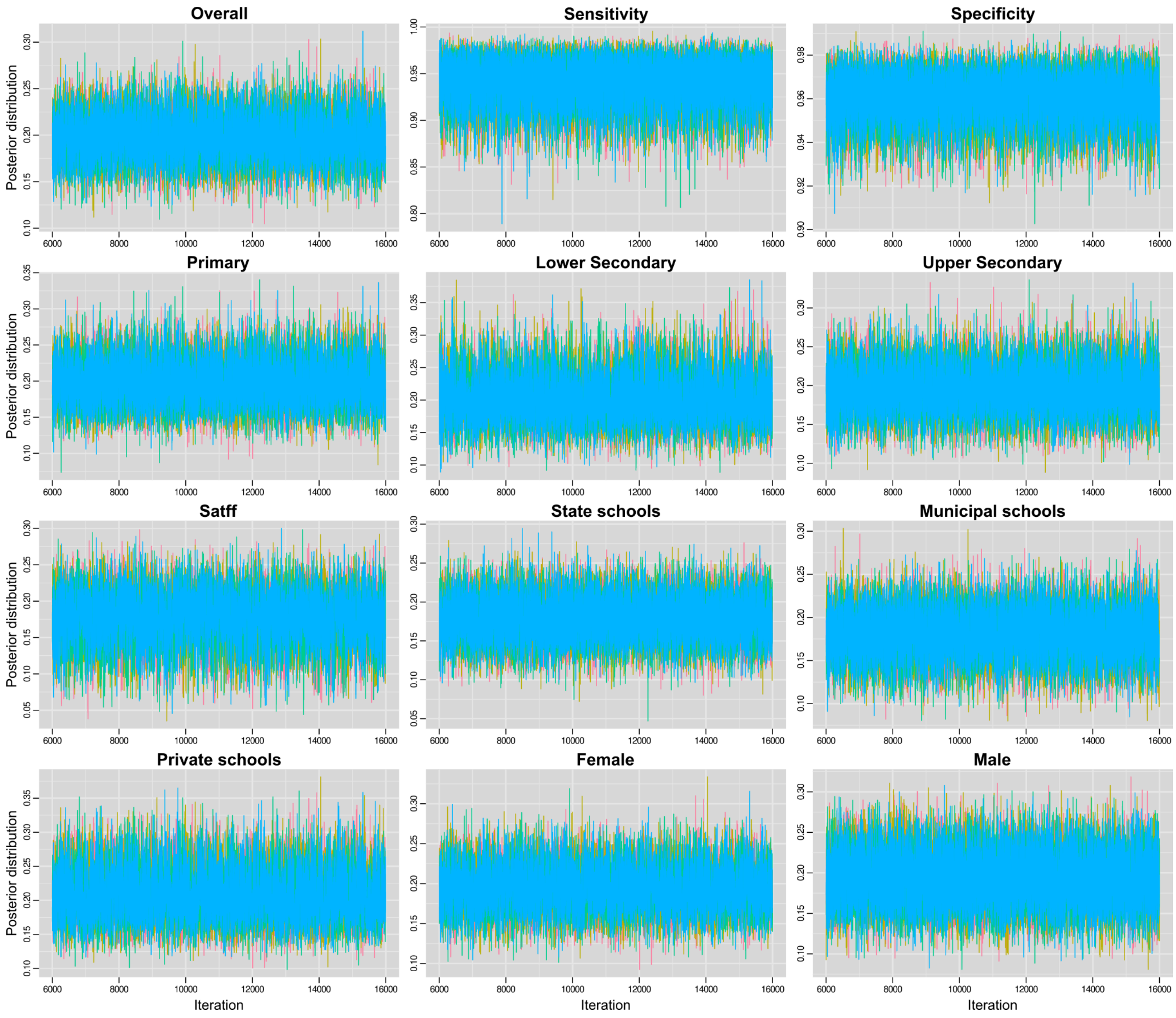

Supplementary Figure 1. Markov chain Monte Carlo (MCMC) trace plots for the model from the first test period (from October 18 to December 1, 2021). The plots are presented for the school community (overall), sensitivity and specificity of the immunological test, education degree (primary, lower secondary, upper secondary and staff), school type (municipal, state and private) and sex (female and male). The colors indicate each of the four chains used in the analysis.

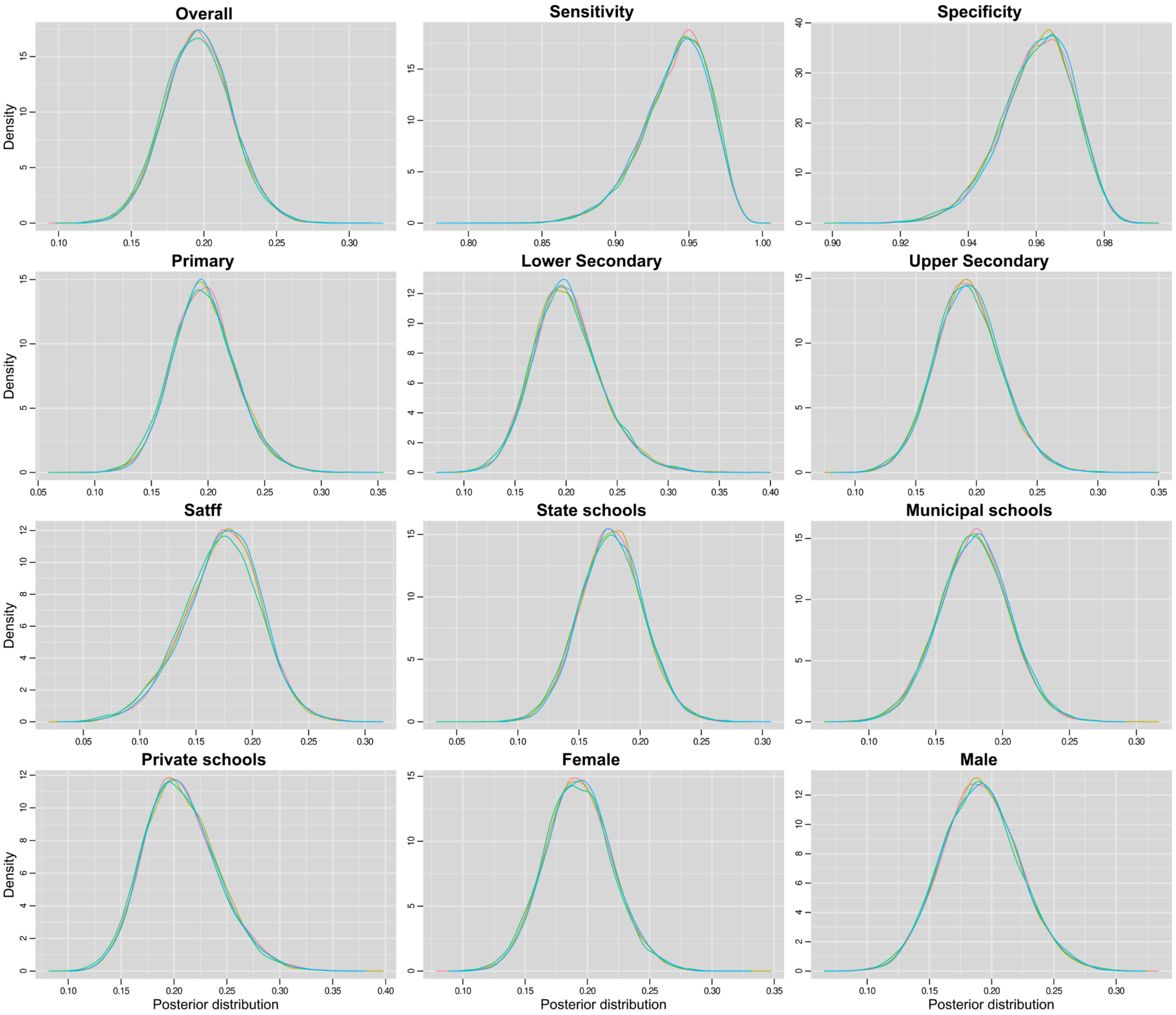

Supplementary Figure 2. Markov chain Monte Carlo (MCMC) density plots for the model from the first testing period (from October 18 to December 1, 2021). The plots are presented for the school community (overall), sensitivity and specificity of the immunological test, education degree (primary, lower secondary, upper secondary and staff), school type (municipal, state and private) and sex (female and male). The colors indicate each of the four chains used in the analysis.

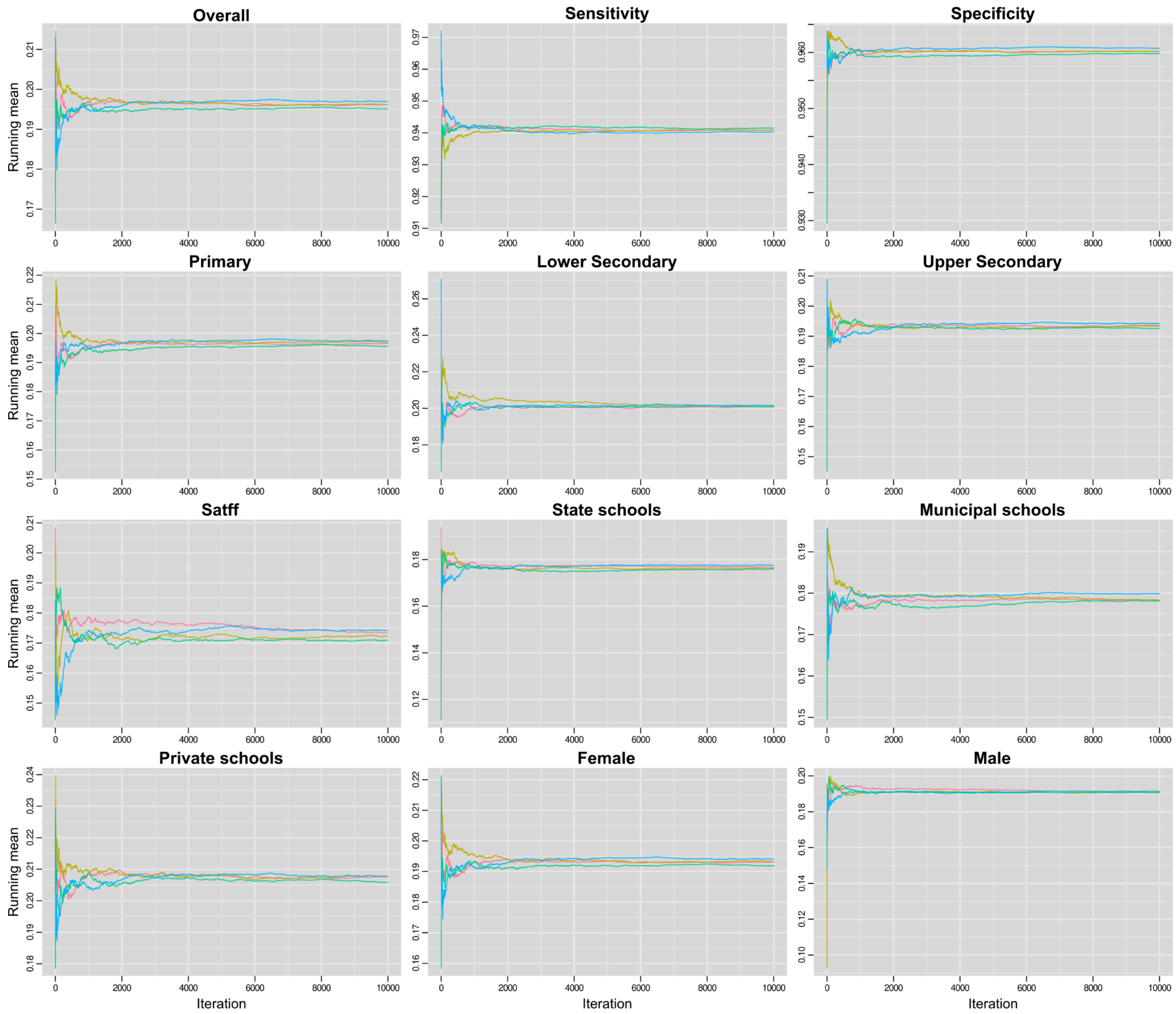

Supplementary Figure 3. Markov chain Monte Carlo (MCMC) running mean plots for the model from the first testing period (from October 18 to December 1, 2021). The plots are presented for the school community (overall), sensitivity and specificity of the immunological test, education degree (primary, lower secondary, upper secondary and staff), school type (municipal, state and private) and sex (female and male). The colors indicate each of the four chains used in the analysis.

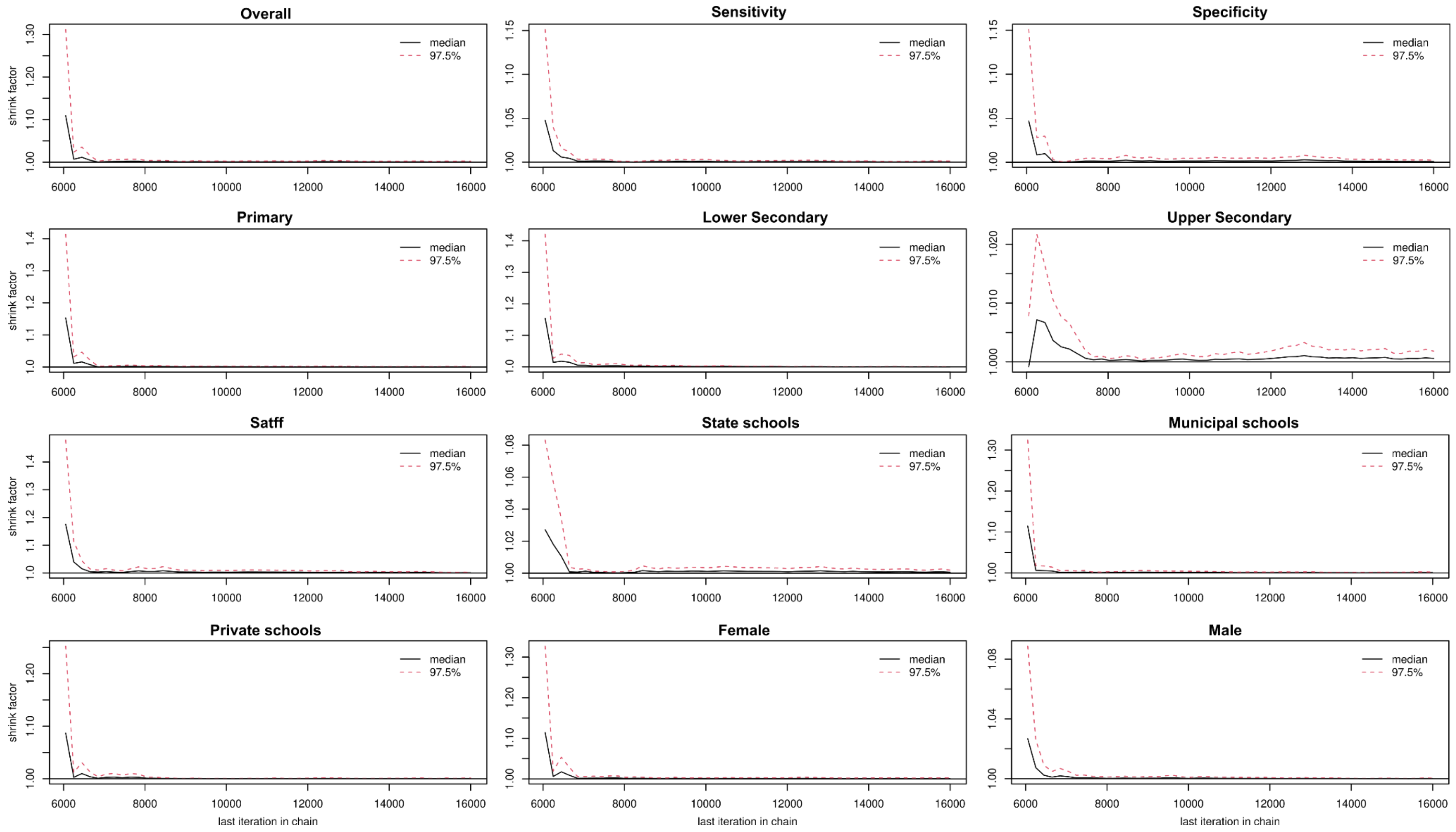

Supplementary Figure 4. Plots of the evolution of Gelman and Rubin's shrink factor as the number of iterations increases for the model from the first testing period (from October 18 to December 1, 2021). The plots are presented for the school community (overall), sensitivity and specificity of the immunological test, education degree (primary, lower secondary, upper secondary and staff), school type (municipal, state and private) and sex (female and male).

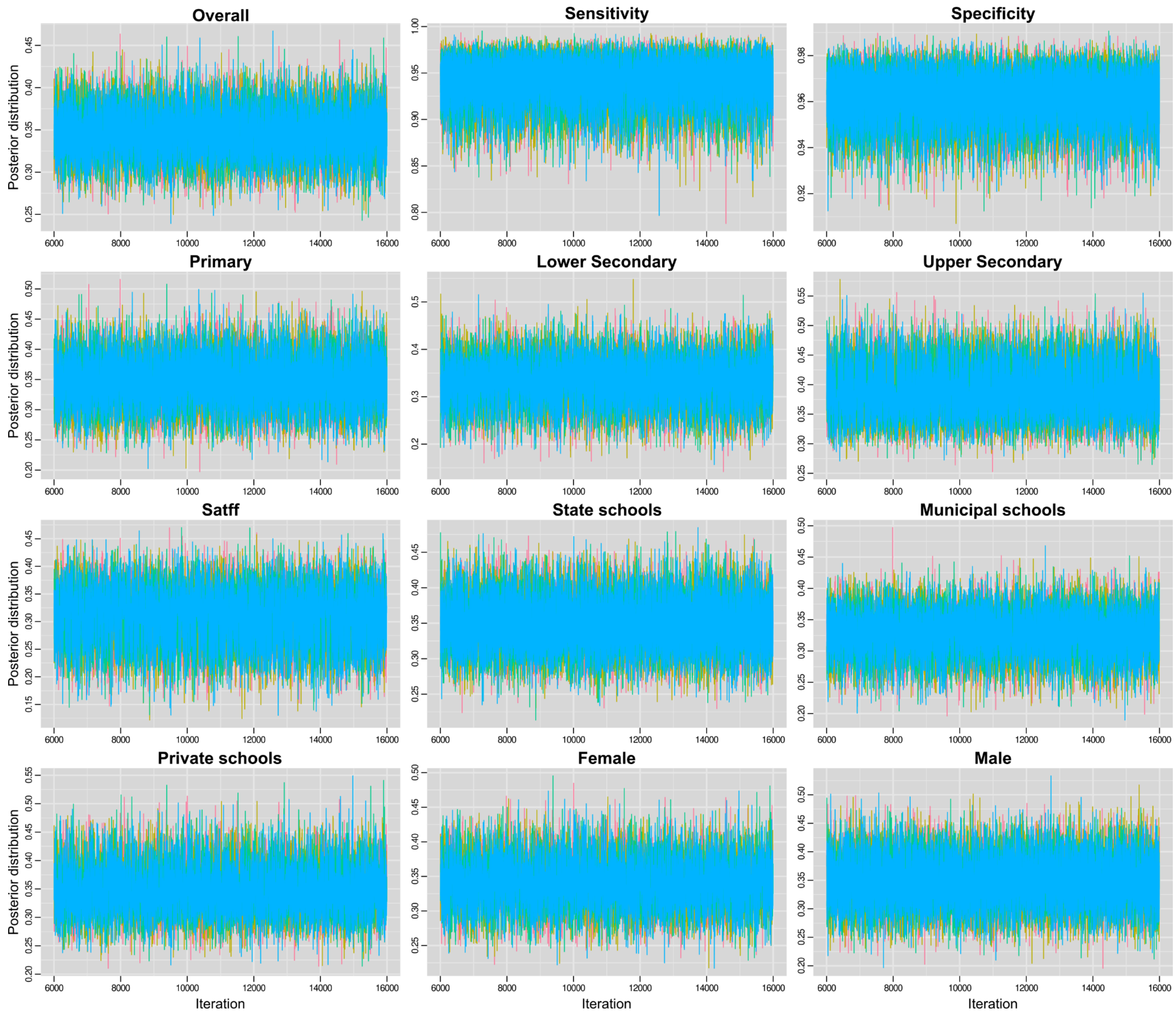

Supplementary Figure 5. Markov chain Monte Carlo (MCMC) trace plots for the model from the second testing period (from March 9 to July 4, 2022). The plots are presented for the school community (overall), sensitivity and specificity of the immunological test, education degree (primary, lower secondary, upper secondary and staff), school type (municipal, state and private) and sex (female and male). The colors indicate each of the four chains used in the analysis.

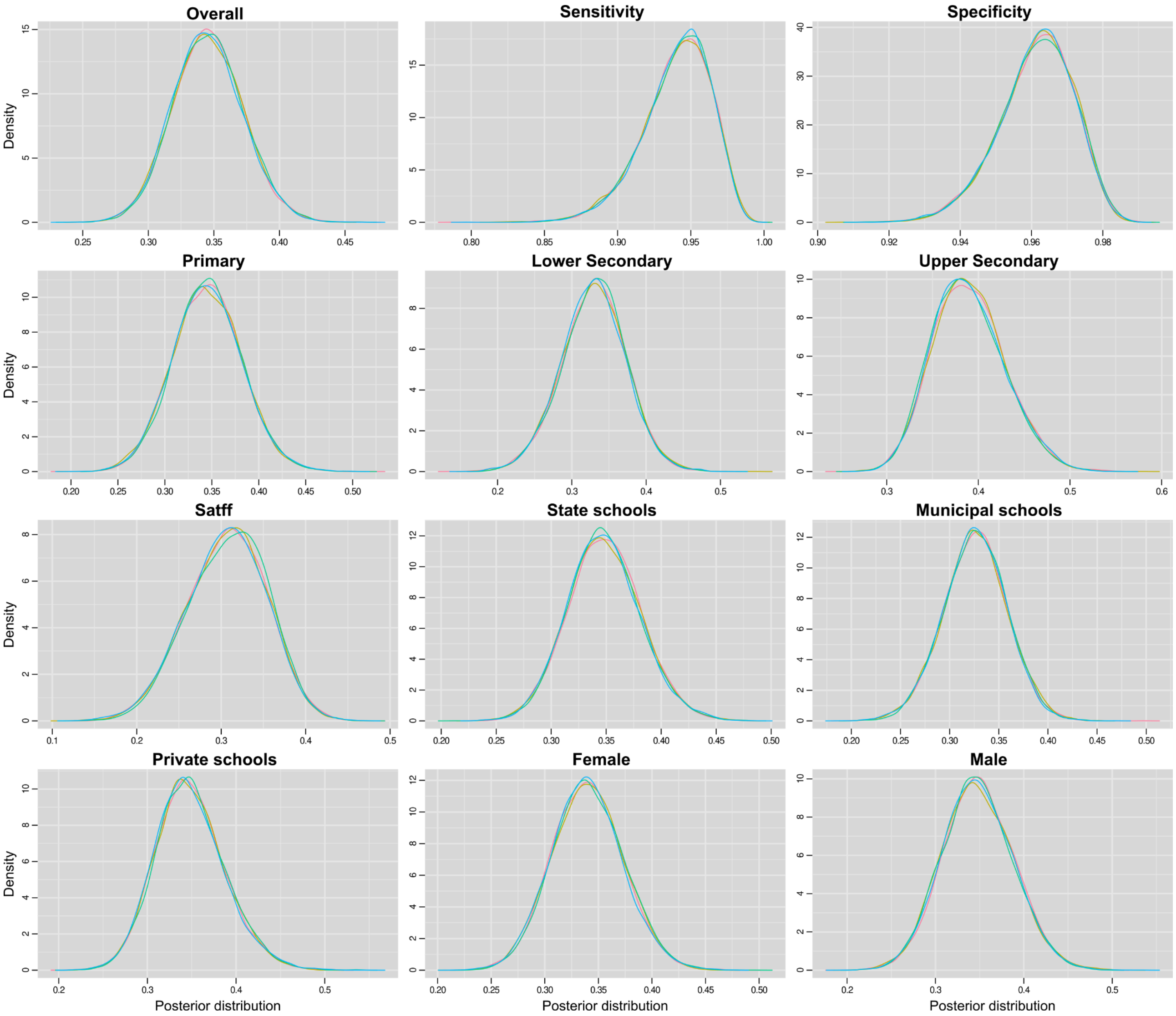

Supplementary Figure 6. Markov chain Monte Carlo (MCMC) density plots for the model from the second testing period (from March 9 to July 4, 2022). The plots are presented for the school community (overall), sensitivity and specificity of the immunological test, education degree (primary, lower secondary, upper secondary and staff), school type (municipal, state and private) and sex (female and male). The colors indicate each of the four chains used in the analysis.

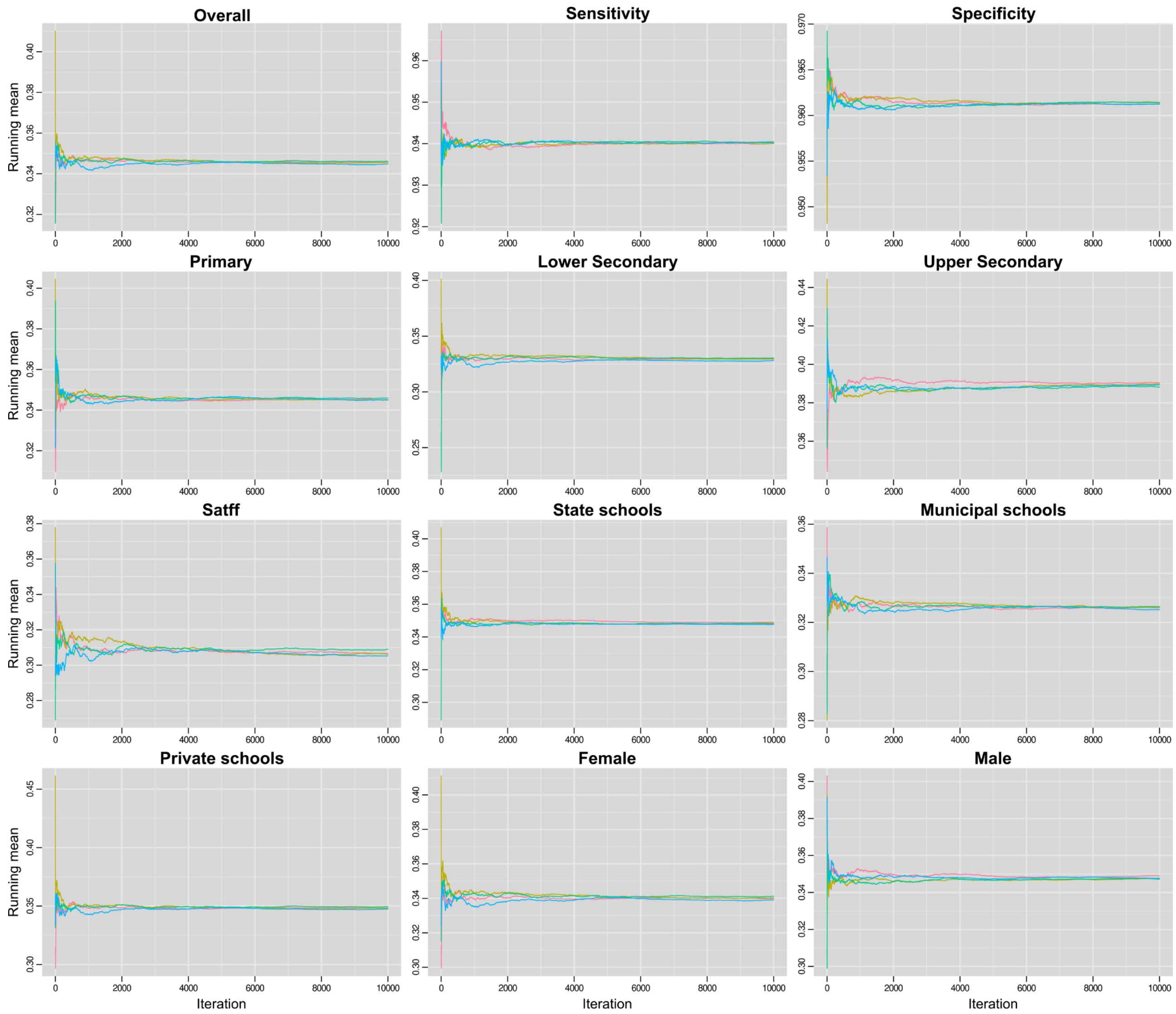

Supplementary Figure 7. Markov chain Monte Carlo (MCMC) running mean plots for the model from the second testing period (from March 9 to July 4, 2022). The plots are presented for the school community (overall), sensitivity and specificity of the immunological test, education degree (primary, lower secondary, upper secondary and staff), school type (municipal, state and private) and sex (female and male). The colors indicate each of the four chains used in the analysis.

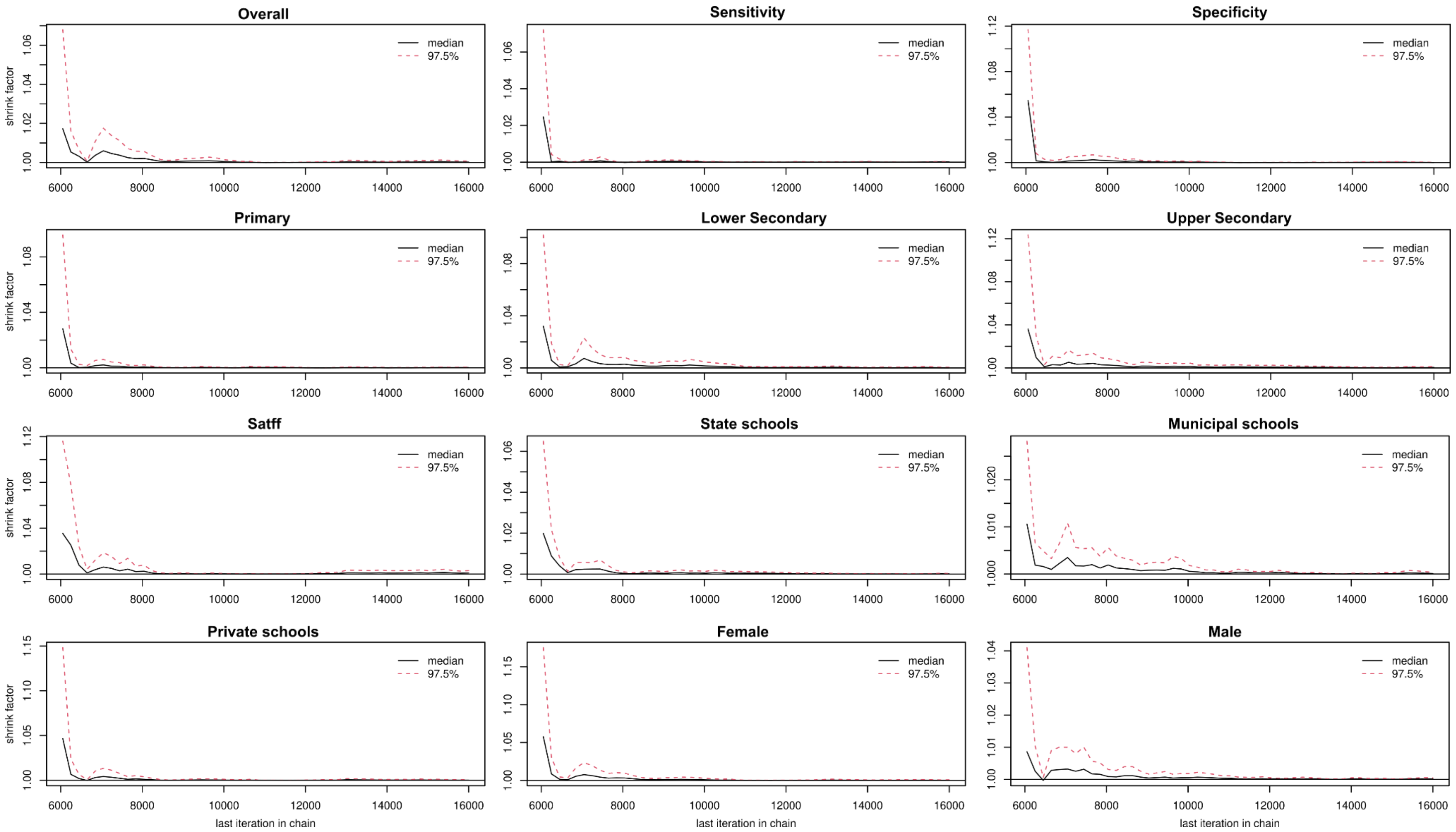

Supplementary Figure 8. Plots of the evolution of Gelman and Rubin's shrink factor as the number of iterations increases for the model from the second testing period (from March 9 to July 4, 2022). The plots are presented for the school community (overall), sensitivity and specificity of the immunological test, education degree (primary, lower secondary, upper secondary and staff), school type (municipal, state and private) and sex (female and male).

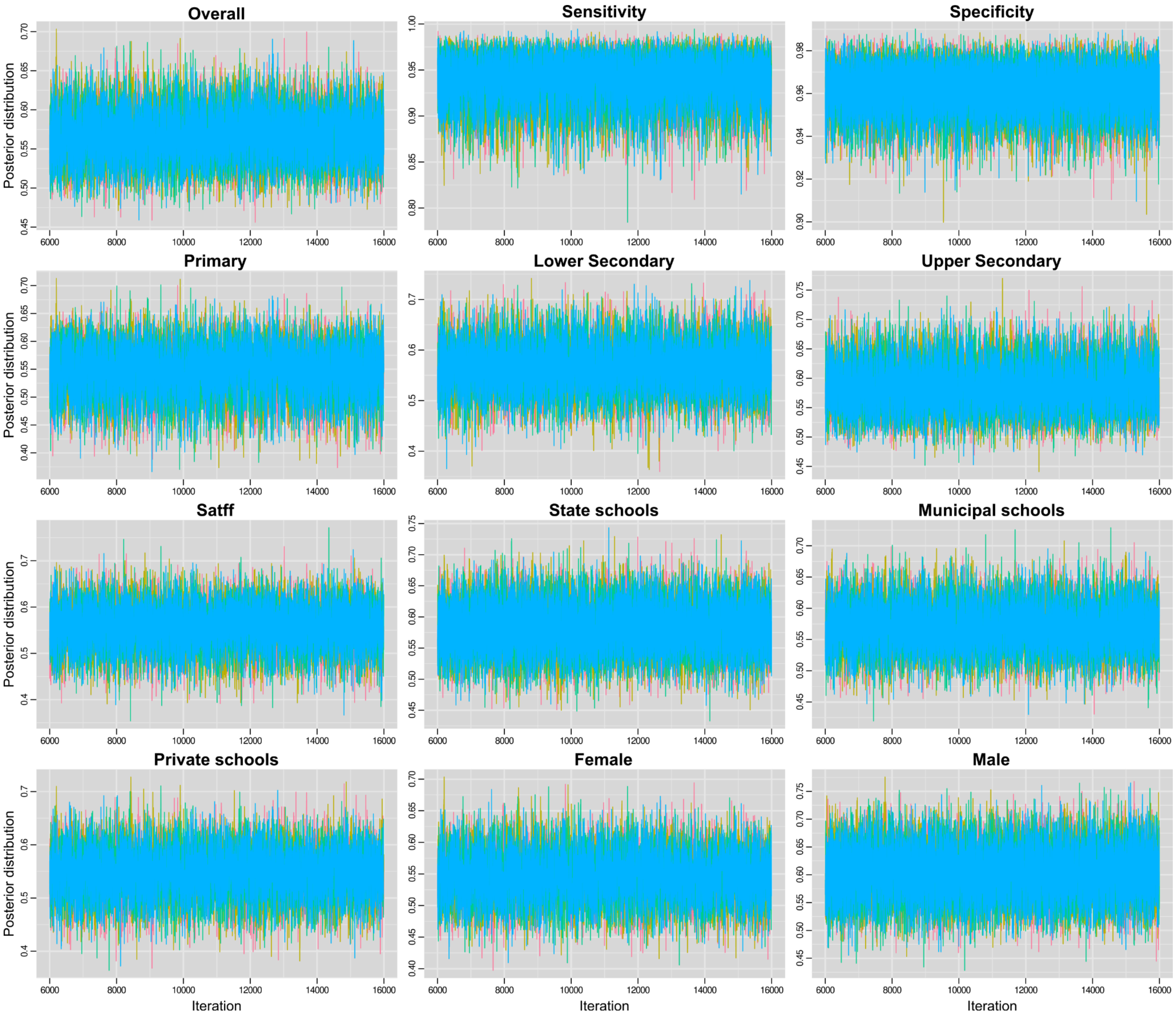

Supplementary Figure 9. Markov chain Monte Carlo (MCMC) trace plots for the model from the third testing period (from August 2 to November 21, 2022). The plots are presented for the school community (overall), sensitivity and specificity of the immunological test, education degree (primary, lower secondary, upper secondary and staff), school type (municipal, state and private) and sex (female and male). The colors indicate each of the four chains used in the analysis.

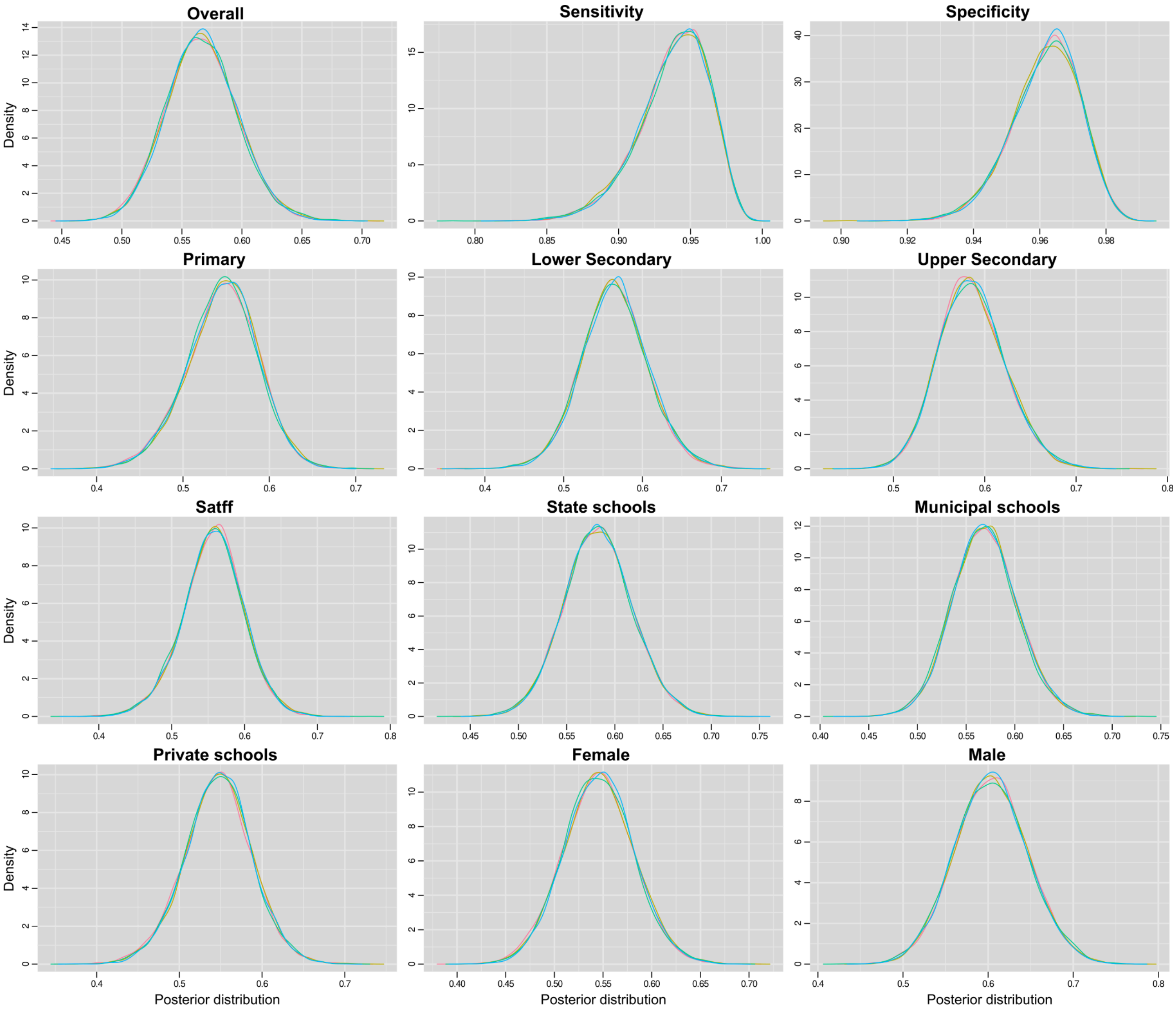

Supplementary Figure 10. Markov chain Monte Carlo (MCMC) density plots for the model from the third testing period (from August 2 to November 21, 2022). The plots are presented for the school community (overall), sensitivity and specificity of the immunological test, education degree (primary, lower secondary, upper secondary and staff), school type (municipal, state and private) and sex (female and male). The colors indicate each of the four chains used in the analysis.

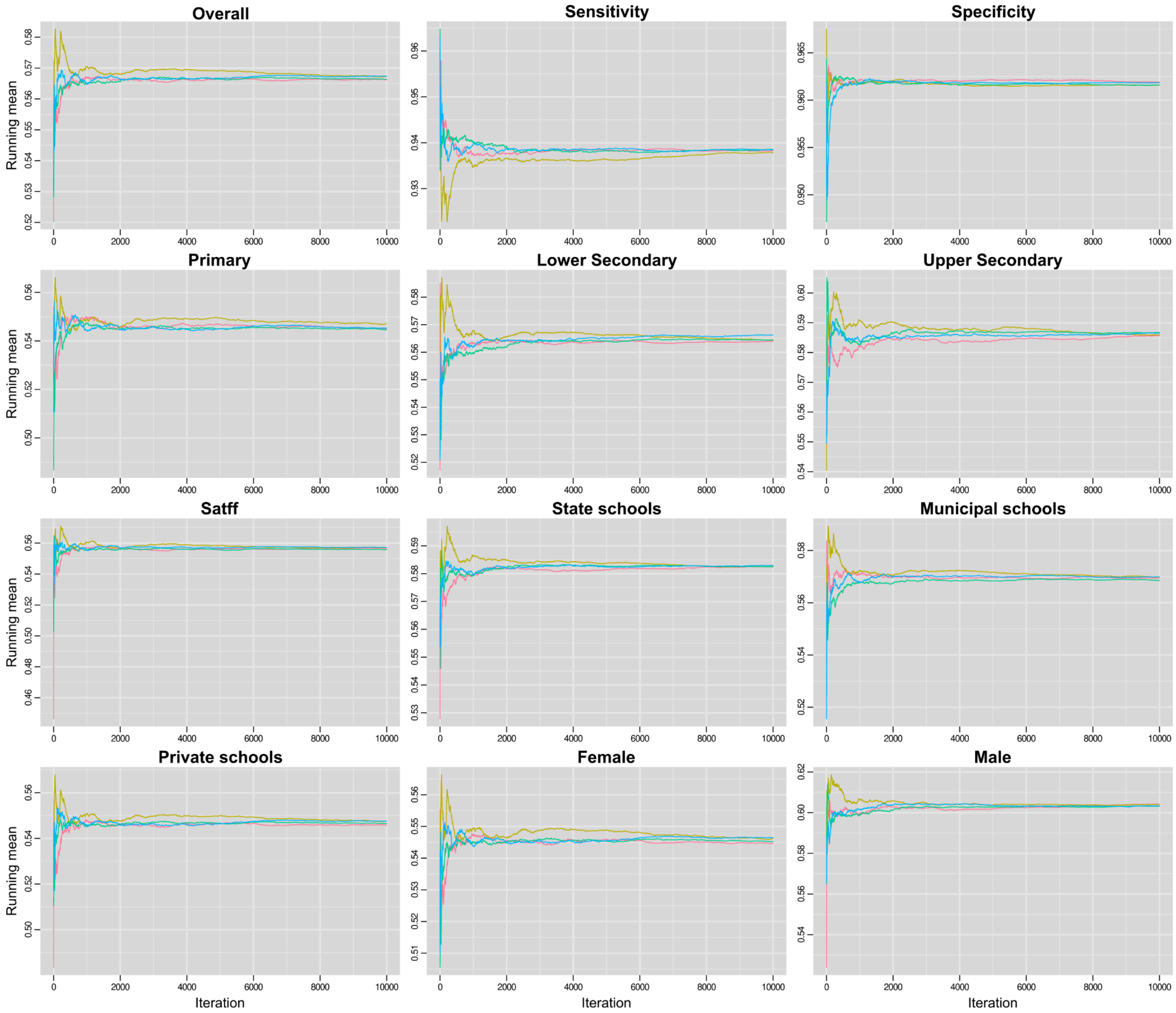

Supplementary Figure 11. Markov chain Monte Carlo (MCMC) running mean plots for the model from the third testing period (from August 2 to November 21, 2022). The plots are presented for the school community (overall), sensitivity and specificity of the immunological test, education degree (primary, lower secondary, upper secondary and staff), school type (municipal, state and private) and sex (female and male). The colors indicate each of the four chains used in the analysis.

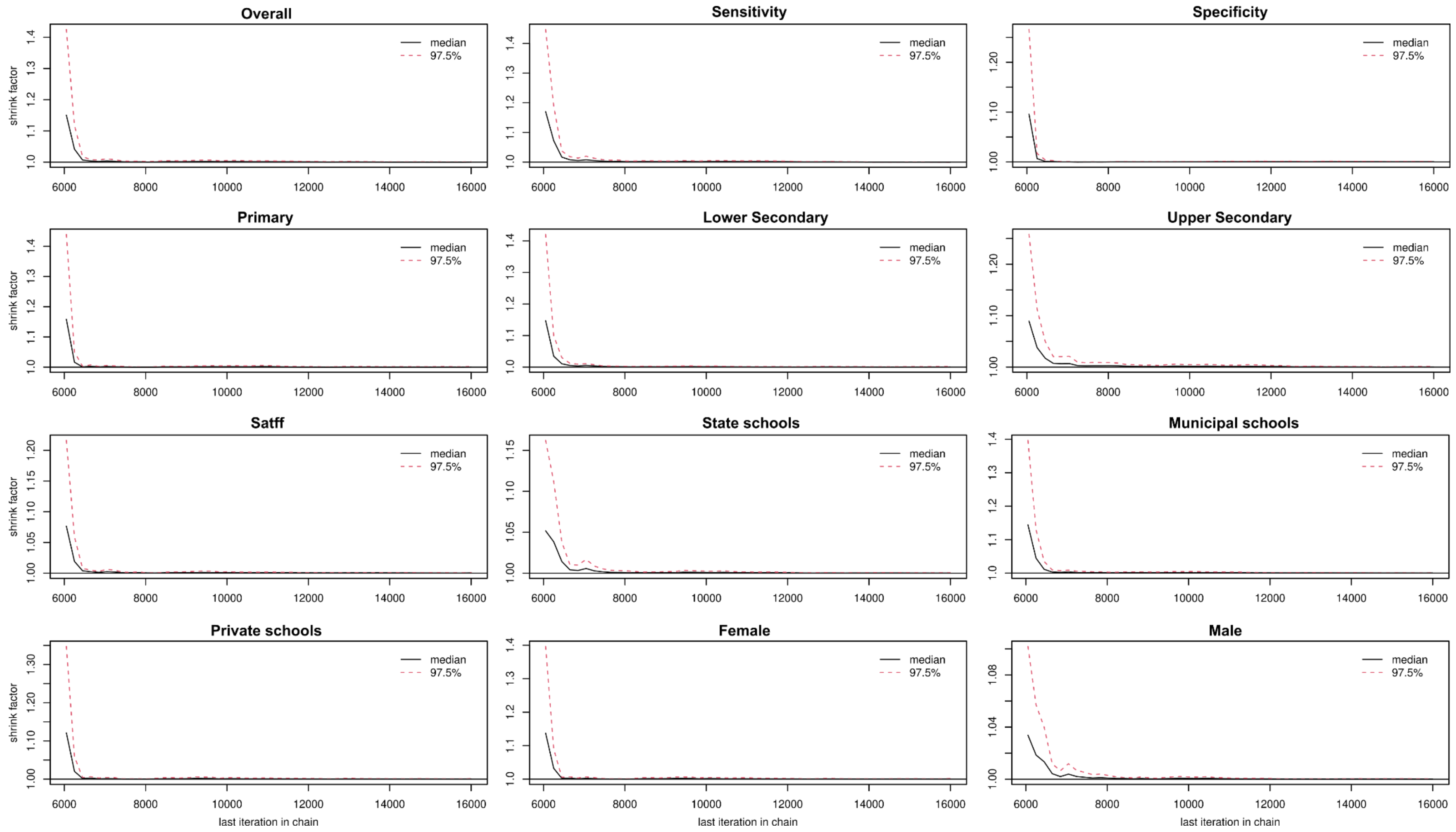

Supplementary Figure 12. Plots of the evolution of Gelman and Rubin's shrink factor as the number of iterations increases for the model from the third testing period (from August 2 to November 21, 2022). The plots are presented for the school community (overall), sensitivity and specificity of the immunological test, education degree (primary, lower secondary, upper secondary and staff), school type (municipal, state and private) and sex (female and male).
